# Population Physiologically‐Based Pharmacokinetic Modeling to Determine Ontogeny: A Quantitative Clinical Pharmacology Example in Pediatric Rare Disease

**DOI:** 10.1002/psp4.70174

**Published:** 2026-01-29

**Authors:** Yumi Cleary, Bhagwat Prasad, Kayode Ogungbenro, Michael Gertz, Aleksandra Galetin

**Affiliations:** ^1^ Centre for Applied Pharmacokinetic Research, Division of Pharmacy and Optometry, School of Health Sciences University of Manchester Manchester UK; ^2^ Roche Pharma Research and Early Development Pharmaceutical Sciences, Roche Innovation Center Basel Basel Switzerland; ^3^ Division of Translational and Clinical Pharmacology Cincinnati Children's Hospital Medical Center Cincinnati Ohio USA

**Keywords:** ontogeny, pediatrics, pharmacometrics, physiologically‐based pharmacokinetic, rare disease

## Abstract

Pediatric physiologically‐based pharmacokinetic (PBPK) modelling plays an increasing role in selecting doses in children and addressing clinical pharmacology questions. Ethical concerns often limit clinical pharmacology studies that have no direct therapeutic benefit in children, highlighting the value of PBPK model predictions. However, regulatory acceptance of pediatric PBPK models remains limited because of uncertainties in system‐specific information and inadequate model qualification. Ambiguous ontogeny data of drug metabolizing enzymes (DME) and transporters are recognized as significant obstacles to the accurate pharmacokinetics (PK) prediction in children and the leading cause of insufficient pediatric PBPK model qualification. To address this challenge, a population PBPK modeling approach is proposed. This method is analogous to whole‐body PBPK modeling and allows the estimation of DME/transporter ontogenies using sparse PK data collected from children and adults by nonlinear mixed‐effect modeling. Well‐characterized ontogeny functions of key DME/transporters enhance the extrapolation ability of PBPK models and facilitate model‐informed drug development (MIDD) in children. This article proposes a strategy for pediatric PK extrapolation using population PBPK modeling, illustrated through the case example of risdiplam, approved for the treatment of spinal muscular atrophy. The ontogeny modeling, extrapolations of PK to unstudied pediatric populations, and drug–drug interaction (DDI) risk assessment are also discussed. The population PBPK modeling approach is intended to address the inconsistencies in ontogeny data and augment PBPK modeling for quantitative clinical pharmacology assessments in children. It will accelerate optimal dose finding and provide guidance for adequate use of drugs in pediatric patients, which is especially important for developing treatments for progressive pediatric rare diseases.

## 1 Introduction

The prediction of pharmacokinetics (PK) in children is a fundamental step in pediatric drug development. Maintaining the target exposure is a prerequisite for pediatric trials to avoid subtherapeutic exposure, as emphasized in the ICH E11A guideline. This is particularly critical for pediatric patients suffering from rare diseases, which are often progressive, life‐threatening, and have limited treatment options. Whole‐body physiologically‐based pharmacokinetic (PBPK) modeling facilitates the translation of preclinical/in vitro study outcomes and PK data obtained in adult populations to children. Besides PK predictions and dose selection, whole‐body PBPK modeling enables quantitative clinical pharmacology evaluations in children, such as drug–drug interaction (DDI), the effects of food on absorption, and genetic polymorphisms on PK [1]. Whole‐body PBPK modeling can guide the appropriate use of medicines in pediatric patients without conducting clinical investigations, which are often without clear therapeutic benefit and are avoided due to ethical consideration. The use of pediatric whole‐body PBPK modeling to inform drug labels in lieu of clinical trials is emerging [2] and, therefore, its importance in pediatric drug development is expected to increase.

**FIGURE 1:**
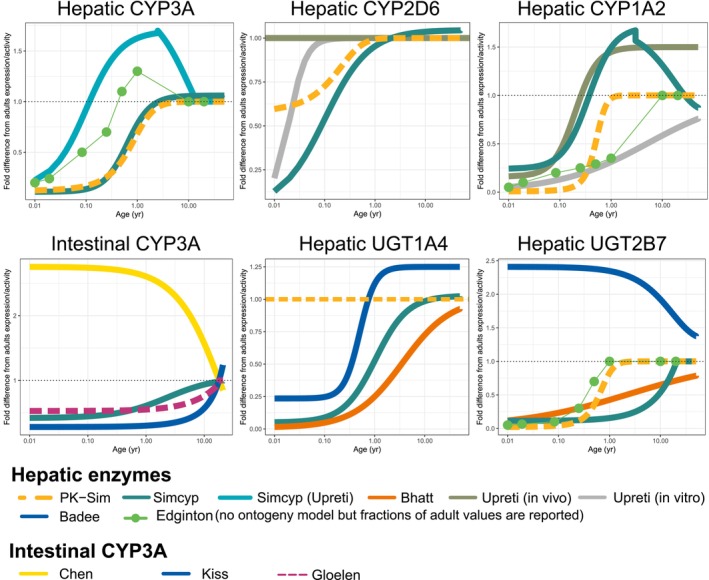
Ontogeny models for drug metabolizing enzymes. Ontogeny models/data of typical DMEs, hepatic CYP1A2, CYP2D6 and CYP3A and intestinal CYP3A, hepatic UGT1A4 and UGT2B7. The references of each ontogeny model or data are provided in Table S1. Two ontogeny functions of hepatic CYP3A of Simcyp Profile 1 (Salem et al. [6]) and Profile 2 (Upreti et al. [7] with modification according to [8]) are shown.

While whole‐body PBPK modeling offers diverse extrapolation opportunities, uncertainties in system data and insufficient qualification of the models often hinder reliable extrapolations and regulatory acceptance of pediatric whole‐body PBPK model applications [3, 4]. Refining age‐dependent physiology and drug‐specific biochemical parameters, along with a better understanding of drug metabolizing enzyme (DME) and transporter ontogeny, is key to improving the predictive performance and regulatory acceptance of pediatric whole‐body PBPK models [1, 5]. Evaluations of ontogeny and age‐dependent changes in the quantitative contributions of the various elimination pathways are also required in the EMA PBPK modelling guideline. Although significant advancement in ontogeny research was made in the last two decades, reports for the same DME are often ambiguous, as shown in Figure 1. Differences in in vitro ontogeny study reports may stem from variable pediatric sample sizes, unbalanced age distributions, the quality of the tissue affected by post‐mortem intervals and tissue preservation or underlying disease of the donors [9]. Endogenous biomarkers may be used for in vivo ontogeny assessments considering that several biomarkers are currently available for hepatic and renal transporters [10, 11]. However, these have not been explored for ontogeny purposes so far. In vivo ontogeny functions have been mostly derived by the relative difference in deconvoluted hepatic intrinsic clearance (CL_int_) values from clinical PK data using a well‐stirred liver model accounting for age‐dependent anatomical and physiological data (e.g., microsomal protein [MPPGL], blood flow) between adults and children [6, 7]. The success of this approach is also dependent on various factors. Sparse sample collection in children may greatly reduce the confidence in the estimated clearance (CL) values (unless based on population approaches). In addition, study design (age range, sample size), disease, and assumptions made for age‐dependent physiology in modeling may introduce bias in the estimation of the ontogeny function. The reliability of the estimated ontogeny function needs to be evaluated in terms of the adequacy of selected structural models, precision of parameter estimates, and overall prediction ability including the central tendency and variability of the observations in children. However, such assessments are rarely discussed in the reported analyses.

Inconsistent ontogeny data present a major challenge for reliable PK predictions in children [5]. The consequence of inconsistent enzyme ontogeny was demonstrated in more than twofold differences in tramadol CL predictions in neonates [12] mainly due to different CYP2D6 ontogeny (Figure 1) and MPPGL. Similarly, up to twofold differences in ivabradine and metabolite AUC (area under concentration‐time curve) were predicted in children 0.5–3 years old between the Salem and the Upreti functions for hepatic CYP3A4 ontogeny (Figure 1), leading to 1.4‐fold differences in predicted heart rates [13]. Despite efforts to standardize approaches [5], currently there is no consensus nor guidance on the selection or handling of inconsistent ontogeny data.

## 2 Application of Population PBPK Modeling for In Vivo Ontogeny Estimations

Population PK (PPK) and whole‐body PBPK modeling are often conducted in parallel during pediatric drug development [14]. Blood samples collected in children are usually sparse, and PPK modeling is vital to estimate reliable individual PK parameters (AUC, CL). While such estimates are often used to evaluate the prediction ability of pediatric whole‐body PBPK models, they do not directly evaluate the assumed ontogeny models. The use of whole‐body PBPK models for parameter estimation has to overcome challenges of overparameterization within a complex model structure, structural and practical identifiability, and significant computation time [15]. Alternatively, population PBPK models have been used as an integration of PPK and whole‐body PBPK modeling. These models have reduced model structure but retain physiological complexity in organs that are relevant for the modeling objective [15]. This approach has been applied to address complex DME and transporter‐mediated disposition or DDI‐related questions [15, 16]. Population PBPK modeling intended to estimate DME/transporter ontogeny functions has been applied for the ontogeny of hepatic and intestinal CYP3A enzymes using midazolam [17], and OAT1/3 transporters using clavulanic acid and amoxicillin [18] data. In industry, population PBPK modeling was conducted for risdiplam (Evrysdi) to estimate in vivo FMO3 ontogeny to address pediatric quantitative clinical pharmacology questions to support its development for the treatment of spinal muscular atrophy (SMA) [19]. The risdiplam case example is briefly illustrated here.

## 3 A Case Example of Risdiplam's Clinical Development in Patients With Spinal Muscular Atrophy

**FIGURE 2:**
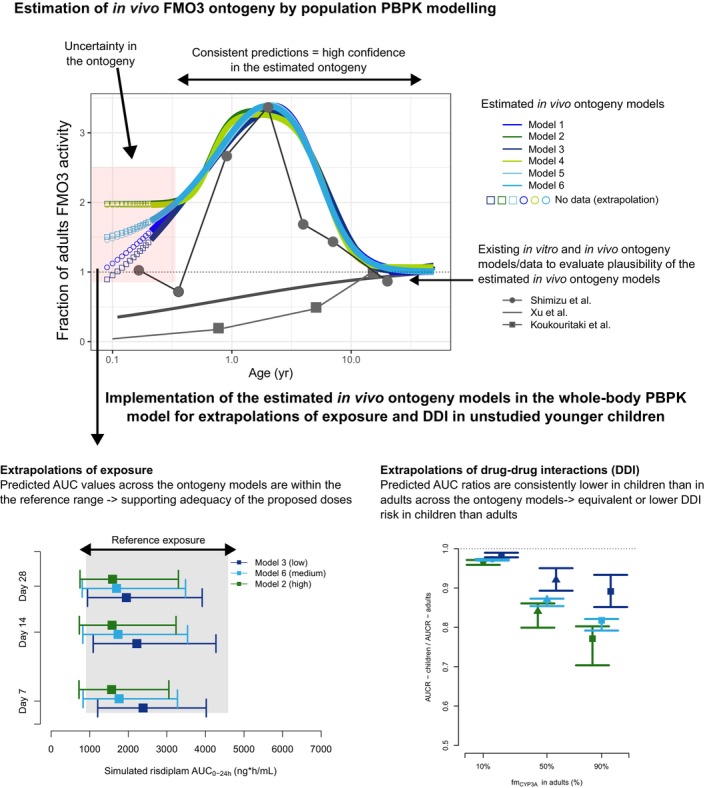
Proposed strategy for pediatric PK extrapolations using population PBPK modeling for quantitative clinical pharmacology assessments in pediatric drug development. The example is based on risdiplam case study [19]. The in vivo FMO3 ontogeny was estimated by population PBPK modeling of risdiplam data in patients with SMA including 382 pediatric patients aged 2 months to 18 years, with whom PK was evaluated for the median duration of 439 days and up to 3 years. Six structural models were examined for FMO3 ontogeny and all ontogeny models equally and adequately predicted risdiplam PK across the age range. Good precision of parameter estimates was supported by bootstrap analyses, goodness‐of‐fit plots and visual predictive check indicated adequacy of the estimated FMO3 ontogeny [19]. All estimated FMO3 ontogeny model predictions were consistent in the age group of ≥ 4 months old. The estimated models were supported by the independent in vitro study of FMO3 ontogeny where trimethylamine N‐oxygenation was measured in the liver microsomes (*n* = 9, aged 13 days to 7 years old) [21]. Since variable FMO3 trajectories were predicted in the age range of < 4 months old, three of the estimated ontogeny models predicting the lowest, medium and highest FMO3 activity/expressions were chosen for the extrapolations in this age range. One example of extrapolation was prediction of risdiplam AUC in 16‐day old neonates to evaluate the adequacy of 0.15 mg/kg dose. The predicted AUC values differ among the ontogeny models; however, they were all mostly within the range of risdiplam exposure observed in the older pediatric patients treated with the approved dose. Another example was predictions of CYP3A substrate DDI propensity of dual CYP3A‐FMO3 substrates in children 2–4 months old [19]. The predicted AUC ratios of the dual substrates in the absence/presence of potent CYP3A inhibitors were equivalent or lower than adults across the ontogeny models, indicating lower CYP3A4 DDI propensity in children for dual FMO3‐CYP3A4 substrates. This is a data‐driven approach to quantify uncertainty of ontogeny directly in the target patient population and complements conventional sensitivity analyses with a range of ontogeny functions which are mostly studied outside of target populations.

**FIGURE 3:**
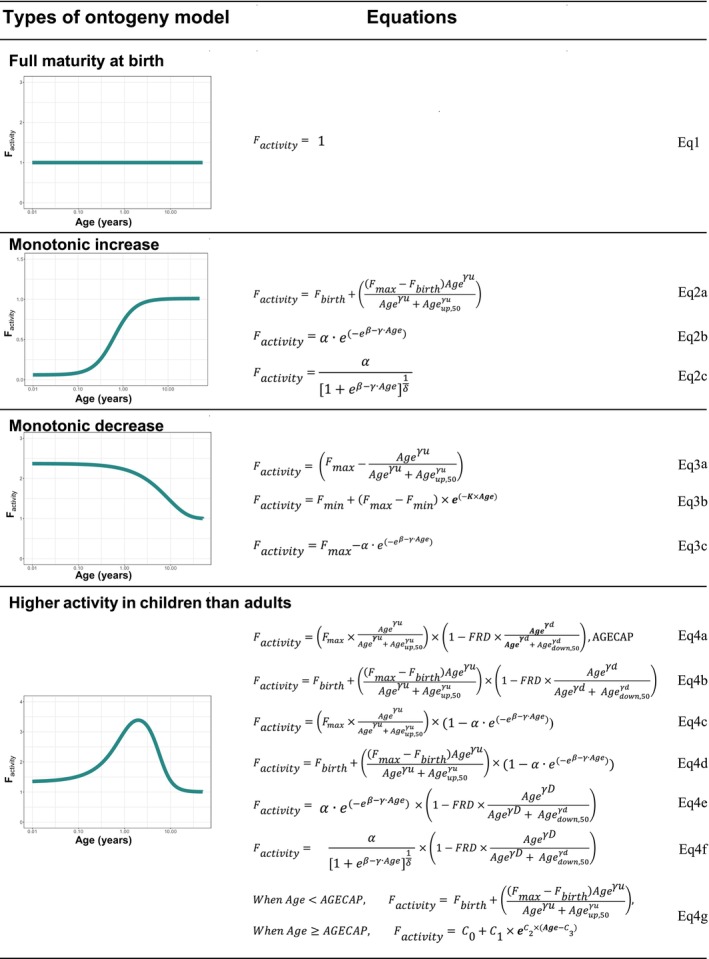
Examples of structural models for DME/transporter ontogeny. For the estimation of in vivo FMO3 ontogeny, the Equations 4a–f in the category of “Higher activity in children than adults” were used. These six models consist of 6–7 parameters whereas the model of Equation 4g used for the ontogeny study [7] requires 9 parameters to estimate. Equation 3b was adapted from [23]. Abbreviations: AGECAP = age to reach the fractional activity = 1, Age_down,50_ = age to reach 50% of the lowest fractional activity, Age_up,50_ = age to reach 50% of the maximum fractional activity, *F*_activity_ = fraction of adult's activity at a given age of a child, *F*_birth_ = fraction of activity at birth, *F*_min_ = minimum fraction of activity, *F*_max_ = maximum fraction of activity, FRD = fractional contribution of down slope, *γd* = hill‐coefficient for the down slope, *γu* = hill‐coefficient for the up slope, *K* = development rate.

SMA is a rare and progressive neuromuscular disease caused by an absence of functional SMN1 gene. Risdiplam is almost completely absorbed after oral administration and eliminated mainly through hepatic metabolism via FMO3 (75%) and CYP3A (20%), and renal excretion (5%) [2, 19]. The ontogeny of hepatic FMO3 is critical to the prediction of pediatric PK, and three in vitro FMO3 ontogeny reports were available prior to the pediatric study [20, 21, 22]. However, neither of these functions was verified with in vivo data of FMO3 substrates in children. These functions differed in their developmental trajectory (Figure 2): one reported an up to fourfold higher FMO3 activity in children (data from *n* = 9 [21]), the others reported a monotonic increase with age (data from *n* = 240 [20] and *n* = 455 [22]). A meta‐analysis of these heterogeneous reports resulted in a monotonic FMO3 ontogeny function reflecting the large sample size of the latter two studies (Figure S1). The application of this function in a prospective pediatric whole‐body PBPK model of risdiplam significantly under‐predicted CL in young children [19]. Subsequently, risdiplam data collected from 525 subjects aged between 2 months and 61 years were used to estimate in vivo FMO3 ontogeny by population PBPK modeling. The model incorporated physiologically based expressions for bioavailability and hepatic CL, with an empirical description of drug absorption and distribution. The drug‐dependent parameters of the risdiplam whole‐body PBPK model (e.g., fm_CYP3A_, fm_FMO3_) verified in adults based on clinical mass‐balance, DDI, and therapeutic studies were scaled to children. Age‐dependent anatomical and physiological data were considered as done in conventional pediatric PBPK modeling. In addition, compatibility between the population PBPK and whole‐body PBPK models was maintained by including consistent drug‐dependent parameterization and using the well‐stirred liver model [19]. Maintaining consistency in the parameterization between population PBPK and whole‐body PBPK models is critical for the application of the estimated ontogeny function for subsequent extrapolations (e.g., DDI predictions) and novel substrates. Six different structural models of FMO3 ontogeny, combinations of various model types (e.g., sigmoidal *E*_max_, the Gompertz functions), were examined to capture higher observed FMO3 activity in children (Figure 3, Equations 4a–f).

All six in vivo FMO3 ontogeny models consistently predicted higher FMO3 metabolic activity in children ≥ 4 months old, reaching a maximum at 2 years of age, with an approximately threefold higher activity compared to adults [19] (Figure 2). All models showed adequate parameter estimates and prediction ability of central tendency and between‐subject variability of risdiplam plasma concentration across the age range [19]. Differences in in vivo FMO3 ontogeny trajectories in infants < 4 months old by the different models are likely attributed to limited observations in this age range. Sparse data collected at the beginning of treatment pose a challenge to detect enzyme maturation, particularly for low hepatic extraction drugs like risdiplam. Nevertheless, the confidence in the estimated in vivo FMO3 ontogeny was high since (1) risdiplam is an appropriate substrate to study hepatic FMO3 ontogeny because of its high bioavailability, low hepatic extraction, and age‐independent plasma protein binding [19], (2) the ontogeny of the two other elimination pathways, hepatic CYP3A and renal elimination, has been well‐studied in vivo [8, 24, 25], (3) all six models showed consistent predictions in the age range of ≥ 4 months old, and (4) functions were in good agreement with an independent in vitro FMO3 ontogeny study with a different FMO3 substrate [21] (Figure 2).

Subsequently, the whole‐body risdiplam PBPK model in children was updated with the estimated in vivo FMO3 ontogeny functions to extrapolate the outcomes of the time‐dependent CYP3A inhibition and CYP3A substrate interactions of risdiplam studied in healthy adults to children. The PBPK modeling work supported a negligible CYP3A‐mediated DDI risk in pediatric SMA patients in the product label [2]. The uncertainty of the in vivo FMO3 ontogeny for children less than 4‐month old needs to be considered when extrapolating to this age range, as illustrated in Figure 2. The lowest, medium and highest FMO3 activity scenarios in that age range were used in the extrapolations of risdiplam exposure in neonates to evaluate the adequacy of proposed risdiplam dose (rational uncertainty assessment). While predicted risdiplam AUC were variable across the ontogeny models, they were mostly within the exposure range observed in the older children treated with the approved risdiplam dose (Figure 2). The proposed FMO3 ontogeny could not be validated against other FMO3 substrates at this time. However, a theoretical DDI risk assessment of dual CYP3A‐FMO3 substrates in children 2–4 months old was performed. The simulated AUC ratios of dual CYP3A‐FMO3 substrates in the absence/presence of potent CYP3A inhibitor indicated lower CYP3A‐mediated DDI propensity in children for dual CYP3A‐FMO3 substrates. Even though the individual AUC and AUC ratio predictions differed among the ontogeny models, they were aligned in the context of exposure‐matching and lower DDI propensity compared with adults. Consistency across the scenarios increases confidence in the decisions to be made for dose selection and DDI risk assessments. Evaluations of the impact of uncertainty on extrapolations are often performed with sensitivity analyses for ranges of input parameters, or ontogeny models/data in literature which are externally studied outside the target patient populations. Estimation of ontogeny directly from clinical data collected from the target pediatric population and application of the range of estimated ontogeny models is proposed as an alternative data‐driven approach to evaluate uncertainty in the prospective predictions in certain age range.

## 4 Proposed Workflow for Population PBPK Modeling in Future Pediatric Drug Development

**FIGURE 4:**
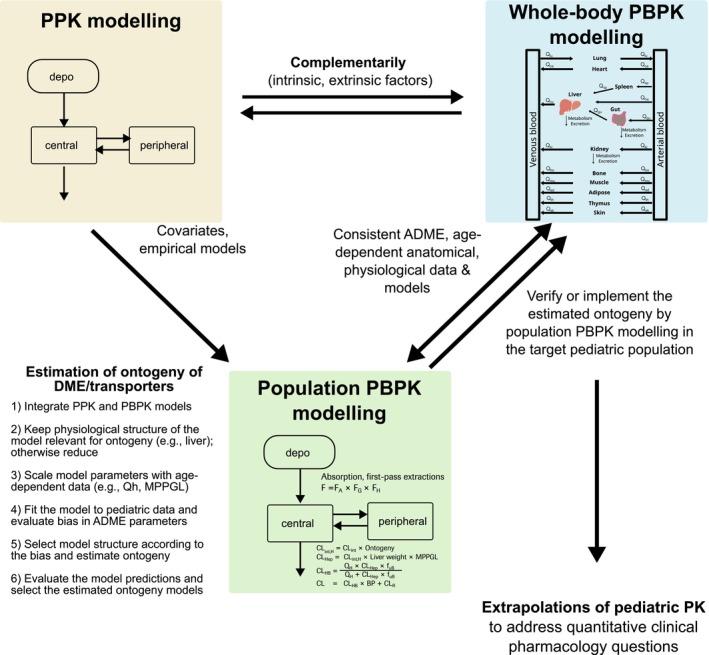
Proposed workflow for pediatric PBPK model‐based extrapolations. PPK and whole‐body PBPK models are often developed in parallel to support pediatric drug development. An integration of PPK and whole‐body PBPK modeling, population‐PBPK modeling, offers population approach to estimate DME/transporter ontogeny from sparse data collected in target pediatric patients. Consistent assumptions in ADME, age‐dependent anatomical and physiological data between the whole‐body and population‐PBPK models are prerequisite for applications of the estimated ontogeny for subsequent extrapolations (e.g., DDI predictions) and to novel substrates.

The proposed workflow for pediatric PK assessments and extrapolations by parallel development of PPK and whole‐body PBPK models, with population PBPK modeling focusing on the assessments of DME/transporter ontogeny, is shown in Figure 4. In the risdiplam example, the population PBPK model implemented a physiologically based hepatic clearance model since FMO3 is expressed in the liver, whereas all other aspects of absorption, distribution, metabolism, and excretion (ADME) were described empirically (irrelevant for the estimation of FMO3 ontogeny). For investigations of the ontogeny of DME/transporters expressed in the intestine or kidney, physiological implementation of intestinal metabolism/renal excretion would be required in the model. The reduction of the model structure of population PBPK models to avoid identifiability issues is particularly important when complex model structures beyond monotonic relationships (Figure 3) are applied for ontogeny. The following steps are recommended for population PBPK modeling to investigate ontogeny in the target pediatric patient populations:

1. Establish a population PBPK model in adults with quantitative ADME parameters (e.g., CL_int_, fractions metabolized [fm] and excreted [fe]) verified with clinical pharmacology study data (human mass‐balance, DDI studies). Reduce the model structure while maintaining the physiological nature of the ADME process(es) involving the DME/transporter of interest. Examine the compatibility with the whole‐body PBPK model.
2. Adapt the population PBPK model developed in adults to children with consideration of age‐dependent anatomical and physiological information (e.g., liver weight, MPPGL) as in the pediatric whole‐body PBPK model. Empirical volume of distribution with relevant covariates can be applied for children with allometric scaling with an exponent of 1.0 when plasma protein binding is age‐independent. Apply the absorption model as defined for adults, unless the whole‐body PBPK model suggests age‐dependent absorption, in which case the population PBPK model should be adapted accordingly. Fit the model to pediatric data and confirm that there is no bias in absorption and volume parameter estimates in children prior to the estimation of DME/transporter ontogeny.
3. When multiple DME/transporters are involved, the choice of ontogeny to estimate depends on the extent of enzyme/transporter contribution to the total clearance and level of uncertainty of the existing ontogeny reports. The ontogeny of hepatic CYP3A has been extensively studied (Table S1). For drugs which partially involve hepatic CYP3A metabolism, and when the majority is eliminated through metabolism of another DME, the hepatic CYP3A ontogeny can be fixed to the well‐established models/data to focus the estimation on the ontogeny of another/less defined DME (e.g., risdiplam example is such a case). However, it is important to note that even for CYP3A enzymes multiple hepatic ontogeny models exist.
4. Examine the relationship between age and post hoc estimates of CL_int_ from the fitting of the population PBPK model without consideration of ontogeny of the DME/transporter of interest to pediatric data. This approach indicates an ontogeny model necessary to describe the age‐CL_int_ relationship. Select existing ontogeny model/data according to the identified relationship and examine the prediction ability of the model in children. When bias is shown, the ontogeny needs to be estimated using clinical data. The recommended practice would be to estimate ontogeny with multiple suitable structural models (Figure 3). Bayesian framework can be considered as an alternative for parameter estimation when there is prior information available, for instance from in vitro data.
5. Evaluate the appropriateness of the population PBPK model including the estimated ontogeny functions by visual and numerical model diagnostics, as typically performed for pharmacometric modeling. When multiple models show equivalent qualifications as illustrated for the risdiplam case, they may be used to represent the uncertainty of the estimated ontogeny for extrapolations outside of the studied age range. Consequently, they should all be retained in future/prospective simulations to address the impact of the uncertainty.
6. Verify or examine plausibility of the estimated ontogeny model using independent in vitro/in vivo ontogeny study data (Figure 2).

## 5 Population PBPK Modeling to Facilitate Model‐Informed Drug Development in Children

Prospective predictions of pediatric PK for starting dose selection in clinical trials require prior ontogeny data/models as parameters for pediatric PBPK models. With the advancement in in vitro and in vivo ontogeny research, the accuracy of prospective pediatric PK prediction is expected to improve. For instance, biomarkers such as creatinine, para‐aminohippuric acid (PAH), and 4‐pyridoxic acid may be considered to assess renal function in vivo. Creatinine reflecting glomerular filtration and PAH and 4‐pyridoxic acid serving as proxies for transporter‐mediated secretion can offer valuable insights into renal filtration and secretion capacity across age groups when they are appropriately validated. However, when a discrepancy between the observations and the prediction is found, the ontogeny of DME/transporters should be estimated or refined by population PBPK modeling using the data collected from the target pediatric patients. The estimated ontogeny model, which demonstrates good precision of parameter estimates and adequate prediction of the central tendency and variability across the age range, should improve qualifications of the pediatric whole‐body PBPK model.

The population PBPK modeling approach is data‐driven and provides an objective assessment of the ontogeny of DME/transporters. Considering the necessity for a population‐based approach to analyze sparse pediatric samples, population PBPK modeling offers an advantage over empirical PPK models by incorporating physiological assumptions for ADME processes and therefore, providing mechanistic insights required for the context of use. Yet population PBPK models can be qualified in the same way as empirical PPK models: bootstrap analysis for precision of parameter estimates, goodness of fit, and visual predictive check for the ability to predict central tendency and variability. This can be another advantage of population PBPK modeling over whole‐body PBPK modeling in terms of the maturity of methods and techniques for regulatory decision making. The ICH M15 guideline on model‐informed drug development (MIDD) provides a framework for MIDD evidence assessment which aims to examine the credibility of the model outcomes by (1) availability of other supportive evidence, (2) consequence of under‐ and over‐predictions (wrong decision) on safety and efficacy, and (3) novelty or maturity of modeling methods and techniques applied for regulatory decision making. The population PBPK modeling method for pediatric PK data is novel in its approach. However, it is technically performed and qualified using the established PPK modeling methods (e.g., software, estimation algorithms, and diagnostics) without requiring specific qualification and validation that whole‐body PBPK modeling platforms have to fulfill. Therefore, population‐PBPK modeling may be considered technically mature for regulatory applications and serves to complement whole‐body PBPK models in order to enhance extrapolation capabilities for the pediatric population.

## 6 Conclusion

Efficacy and safety extrapolation from the reference population to children by exposure matching (ICH E11A guideline) is becoming a mainstream strategy in pediatric drug development as the knowledge of disease progression, pharmacodynamics, and experience in utilization of real‐world data advances. A strategy that implements population‐PBPK modeling methods can address shortcomings in pediatric PBPK modeling associated with inconsistent ontogeny data and increase confidence in pediatric PK predictions. To apply the method for a wide range of pediatric trial scenarios, further improvement and additional method development are required. The empirical approach for selection of structural models for ontogeny as shown for risdiplam example can be automated to improve efficiency. Estimation of ontogeny by population PBPK modeling depends on available data. Extremely sparse data or data collected across a limited age range, often the case in pediatric rare disease trials, may not allow reliable assessment or estimation of DME/transporter ontogeny and therefore, extension of the method and guidance for sparse data is needed.

The population PBPK modeling can contribute to informative pediatric study designing, accelerate dose optimizations and enhance the extrapolation ability of pediatric whole‐body PBPK models. This is particularly important for the development of treatments for pediatric rare diseases where clinical investigation is extremely limited and there is often urgency to achieve therapeutic exposure to prevent disease progressions.

## Funding

The authors have nothing to report.

## Conflicts of Interest

Yumi Cleary and Michael Gertz are employees of F.Hoffmann‐La Roche Ltd.

## Supporting information


**Data S1:** psp470174‐sup‐0001‐supinfo.docx.
