# Supplementary material for: Population Physiologically‐Based Pharmacokinetic Modeling to Determine Ontogeny: A Quantitative Clinical Pharmacology Example in Pediatric Rare Disease
Source: CPT Pharmacometrics Syst Pharmacol. 2026 Jan 29;15(2):e70174. doi: 10.1002/psp4.70174 (PMC12853142; doi:10.1002/psp4.70174)
Supplement: Supplementary file 1 — Data S1: psp470174‐sup‐0001‐supinfo.docx. [file PSP4-15-e70174-s001.docx]

Supplementary Material of Population Physiologically-Based Pharmacokinetic Modelling to Determine Ontogeny: A Quantitative Clinical Pharmacology Example in Pediatric Rare Disease

Yumi Cleary^1,2^, Bhagwat Prasad^3^, Kayode Ogungbenro^1^, Michael Gertz^2^, Aleksandra Galetin^1^

^1^Centre for Applied Pharmacokinetic Research, Division of Pharmacy and Optometry, School of Health Sciences, University of Manchester, Manchester, UK. ^2^Roche Pharma Research and Early Development, Pharmaceutical Sciences, Roche Innovation Center Basel, Switzerland, ^3^Division of Translational and Clinical Pharmacology, Cincinnati Children's Hospital Medical Center, Cincinnati, OH 45229, USA

Corresponding author: Yumi Cleary ([yumi.cleary@roche.com](mailto:yumi.cleary@roche.com))

Grenzacherstrasse 124, CH4070 Basel, Switzerland

+41 61 688 7823

**Conflict of interest.** Yumi Cleary and Michael Gertz are employees of F.Hoffmann-La Roche Ltd.

**Funding information.** There is no funding for this work.

**Keywords.** Pediatrics, physiologically-based pharmacokinetic, ontogeny, pharmacometrics, MIDD

**Summary of ontogeny data/models of the hepatic CYP3A, CYP2D6, CYP1A2, UGT1A4, UGT2B7 and intestinal CYP3A enzymes**

**Table S1. Summary of the ontogeny data/models of the hepatic CYP3A, CYP2D6, CYP1A2, UGT1A4, UGT2B7 and intestinal CYP3A**

| **Enzymes** | **Source** | **References** |
| --- | --- | --- |
| **Hepatic CYP3A** | PK-Sim ver. 7.3 *(in vitro)* | (1) |
|  | Simcyp ver. 22 (profile 1), Salem *et al. (in vivo)* | (2, 3),(4)*,(5)*,(6)*,(7)* |
|  | Simcyp ver. 22 (profile 2), Upreti and Wahlstrom (*in vivo*) modified according to (7) | (3, 8), (4)*,(5)*,(6)*,(7)* |
|  | Edginton et al. (*in vitro* and *in vivo*) | (9) |
| **Hepatic CYP2D6** | PK-Sim ver. 7.3 (*in vitro*) | (1) |
|  | Simcyp ver. 22 (*in vitro*) | (3) , (4)*, (7)* |
|  | Upreti and Wahlstrom (*in vitro* and *in vivo*) | (8) |
| **Hepatic CYP1A2** | PK-Sim ver. 7.3 (*in vitro*) | (1), |
|  | Salem *et al. (in vivo)* | (2), (3), (4)*, (7)* |
|  | Upreti and Wahlstrom (*in vitro* and *in vivo*) | (8), (4)*, (7)* |
|  | Edginton et al. (*in vitro* and *in vivo*) | (9) |
| **Hepatic UGT1A4** | PK-Sim ver. 7.3 (*in vitro*) | (1) |
|  | Simcyp ver. 22 (*in vitro*) | (3) |
|  | Bhatt et al (*in vitro*) | (10, 11) |
|  | Badée et al. (*in vitro*) | (12) |
| **Hepatic UGT2B7** | PK-Sim ver. 7.3 (*in vitro* and *in vivo*) | (1) |
|  | Simcyp ver. 22 (*in vitro* and *in vivo*) | (3) |
|  | Bhatt et al (*in vitro*) | (10, 11) |
|  | Badée et al. (*in vitro*) | (12) |
|  | Edginton et al. (*in vitro* and *in vivo*) | (9) |
| **Intestinal CYP3A** | Simcyp ver. 22, Johnson et al. (*in vitro*) | (13), (14)*,(6)* |
|  | Chen et al. (*in vitro*) | (15), (14)* |
|  | Kiss et al. (*in vitro*) | (16), (14)* |
|  | Gloelen et al. (*in vitro*) | (17), (14)* |

*evaluations of the ontogeny models using clinical PK data in children

FMO3 (flavin-containing mono-oxygenase 3) is highly expressed in the liver and catalyzes the oxidation of a variety of substrates such as trimethylamine, catecholamines (18), and nicotine (19). Three *in vitro* ontogeny studies of FMO3 were reported prior to initiation of the pediatric studies of risdiplam by Koukouritaki *et al.* (20), Shimizu *et al.* (21) and Xu *et al.*(22). Koukouritaki *et al.* (20) reported age-dependent increase in FMO3 expression based on liver data from 240 donors (8 weeks of gestation (foetus) and 18 years old) with approximately 20-fold increase from neonates to adults. Shimizu *et al.* (21) quantified FMO3 expression levels and metabolic activity by measuring trimethylamine N-oxygenation functional activity in human liver microsomes collected from 9 donors aged between 13 days to 7 years old and adults. There was good correlation between the expression and the trimethylamine N-oxygenation functional activity in children. Xu *et al.*(22) investigated the ontogeny of FMO3 enzymes in a large cohort of well-characterised pediatric and adult human liver samples collected from 455 donors aged between 0 and 87 years old. A sigmoidal E_max_ model was fitted to the data to describe the age-dependent increase in FMO3 hepatic abundance with sex and genetic variation as covariates. This model predicted approximately 2.2-fold monotonic increase in FMO3 expression from neonates to 6 years of age. A meta-analysis of these *in vitro* FMO3 ontogeny data resulted in a function which describes monotonic increase in FMO3 activity with age as shown in **Figure S1**.


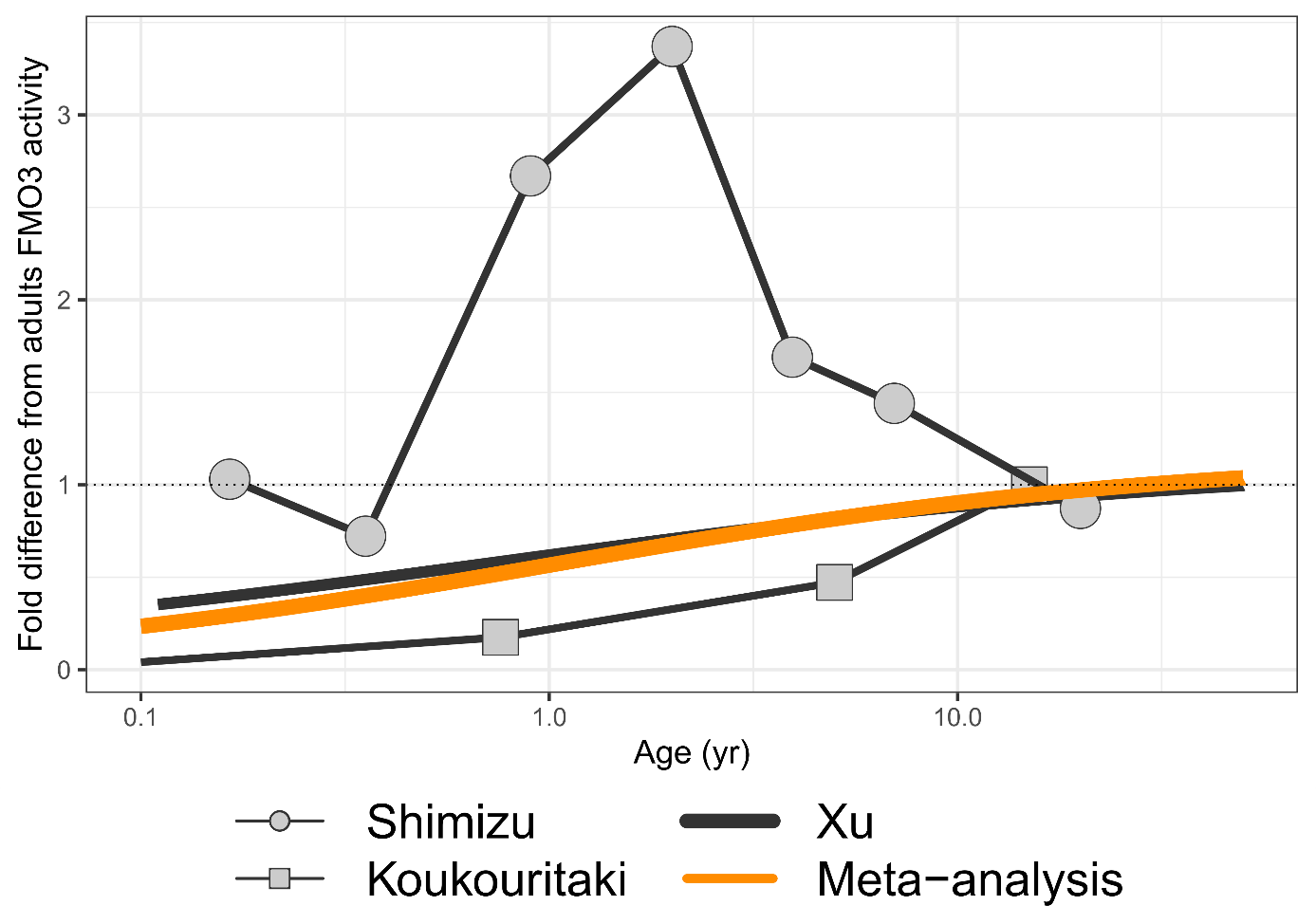


**Figure S1. In vitro FMO3 ontogeny model/data and the FMO3 ontogeny function derived by meta-analysis used for the prospective prediction of risdiplam PK in children**

The trimethylamine N-oxygenation functional activities reported by Shimizu et al. (21) were digitized and normalized by adult value.

**References**

(1) PK-Sim. PK-Sim Ontogeny Database Version 7.3. [*https://githubcom/Open-Systems-Pharmacology/OSPSuiteDocumentation/blob/master/PK-Sim%20Ontogeny%20Database%20Version%2073pdf*](https://githubcom/Open-Systems-Pharmacology/OSPSuiteDocumentation/blob/master/PK-Sim%20Ontogeny%20Database%20Version%2073pdf) **Accessed Februray 2025**, (2018).

(2) Salem, F., Johnson, T.N., Abduljalil, K., Tucker, G.T. & Rostami-Hodjegan, A. A re-evaluation and validation of ontogeny functions for cytochrome P450 1A2 and 3A4 based on in vivo data. *Clin Pharmacokinet* **53**, 625-36 (2014).

(3) Simcyp. Simcyp version 22 user's manual. (2023).

(4) Zhou, W. *et al.* Predictive Performance of Physiologically Based Pharmacokinetic (PBPK) Modeling of Drugs Extensively Metabolized by Major Cytochrome P450s in Children. *Clin Pharmacol Ther* **104**, 188-200 (2018).

(5) Lang, J., Vincent, L., Chenel, M., Ogungbenro, K. & Galetin, A. Impact of Hepatic CYP3A4 Ontogeny Functions on Drug-Drug Interaction Risk in Pediatric Physiologically-Based Pharmacokinetic/Pharmacodynamic Modeling: Critical Literature Review and Ivabradine Case Study. *Clin Pharmacol Ther* **109**, 1618-30 (2021).

(6) Cleary, Y. *et al.* Model-Based Drug-Drug Interaction Extrapolation Strategy From Adults to Children: Risdiplam in Pediatric Patients With Spinal Muscular Atrophy. *Clin Pharmacol Ther* **110**, 1547-57 (2021).

(7) Codaccioni, M., Southall, R.L., Dinh, J. & Johnson, T.N. Prediction of Pediatric Pharmacokinetics for CYP3A4 Metabolized Drugs: Comparison of the Performance of Two Hepatic Ontogeny Within a Physiologically Based Pharmacokinetic Model. *J Clin Pharmacol*, (2024).

(8) Upreti, V.V. & Wahlstrom, J.L. Meta-analysis of hepatic cytochrome P450 ontogeny to underwrite the prediction of pediatric pharmacokinetics using physiologically based pharmacokinetic modeling. *J Clin Pharmacol* **56**, 266-83 (2016).

(9) Edginton, A.N., Schmitt, W., Voith, B. & Willmann, S. A mechanistic approach for the scaling of clearance in children. *Clin Pharmacokinet* **45**, 683-704 (2006).

(10) Bhatt, D.K. *et al.* Hepatic Abundance and Activity of Androgen- and Drug-Metabolizing Enzyme UGT2B17 Are Associated with Genotype, Age, and Sex. *Drug Metab Dispos* **46**, 888-96 (2018).

(11) Bhatt, D.K. *et al.* Age- and Genotype-Dependent Variability in the Protein Abundance and Activity of Six Major Uridine Diphosphate-Glucuronosyltransferases in Human Liver. *Clin Pharmacol Ther* **105**, 131-41 (2019).

(12) Badee, J. *et al.* Characterization of the Ontogeny of Hepatic UDP-Glucuronosyltransferase Enzymes Based on Glucuronidation Activity Measured in Human Liver Microsomes. *J Clin Pharmacol* **59 Suppl 1**, S42-S55 (2019).

(13) Johnson, T.N., Tanner, M.S., Taylor, C.J. & Tucker, G.T. Enterocytic CYP3A4 in a paediatric population: developmental changes and the effect of coeliac disease and cystic fibrosis. *Br J Clin Pharmacol* **51**, 451-60 (2001).

(14) Johnson, T.N., Batchelor, H.K., Goelen, J., Horniblow, R.D. & Dinh, J. Combining data on the bioavailability of midazolam and physiologically-based pharmacokinetic modeling to investigate intestinal CYP3A4 ontogeny. *CPT Pharmacometrics Syst Pharmacol*, (2024).

(15) Chen, Y.T., Trzoss, L., Yang, D. & Yan, B. Ontogenic expression of human carboxylesterase-2 and cytochrome P450 3A4 in liver and duodenum: postnatal surge and organ-dependent regulation. *Toxicology* **330**, 55-61 (2015).

(16) Kiss, M. *et al.* Ontogeny of Small Intestinal Drug Transporters and Metabolizing Enzymes Based on Targeted Quantitative Proteomics. *Drug Metab Dispos* **49**, 1038-46 (2021).

(17) Goelen, J. *et al.* Quantification of drug metabolising enzymes and transporter proteins in the paediatric duodenum via LC-MS/MS proteomics using a QconCAT technique. *Eur J Pharm Biopharm* **191**, 68-77 (2023).

(18) Turkanoglu Ozcelik, A., Can Demirdogen, B., Demirkaya, S. & Adali, O. Flavin containing monooxygenase 3 genetic polymorphisms Glu158Lys and Glu308Gly and their relation to ischemic stroke. *Gene* **521**, 116-21 (2013).

(19) Bloom, A.J., Murphy, S.E., Martinez, M., von Weymarn, L.B., Bierut, L.J. & Goate, A. Effects upon in-vivo nicotine metabolism reveal functional variation in FMO3 associated with cigarette consumption. *Pharmacogenet Genomics* **23**, 62-8 (2013).

(20) Koukouritaki, S.B., Simpson, P., Yeung, C.K., Rettie, A.E. & Hines, R.N. Human hepatic flavin-containing monooxygenases 1 (FMO1) and 3 (FMO3) developmental expression. *Pediatr Res* **51**, 236-43 (2002).

(21) Shimizu, M., Denton, T., Kozono, M., Cashman, J.R., Leeder, J.S. & Yamazaki, H. Developmental variations in metabolic capacity of flavin-containing mono-oxygenase 3 in childhood. *Br J Clin Pharmacol* **71**, 585-91 (2011).

(22) Xu, M. *et al.* Genetic and Nongenetic Factors Associated with Protein Abundance of Flavin-Containing Monooxygenase 3 in Human Liver. *J Pharmacol Exp Ther* **363**, 265-74 (2017).
